# The Impacts of Iron Overload and Ferroptosis on Intestinal Mucosal Homeostasis and Inflammation

**DOI:** 10.3390/ijms232214195

**Published:** 2022-11-17

**Authors:** Caiyun Huo, Guiping Li, Yanxin Hu, Huiling Sun

**Affiliations:** 1 Beijing Key Laboratory for Prevention and Control of Infectious Diseases in Livestock and Poultry, Institute of Animal Husbandry and Veterinary Medicine, Beijing Academy of Agriculture and Forestry Sciences, Beijing 100097, China; 2 Key Laboratory of Animal Epidemiology of Ministry of Agriculture, College of Veterinary Medicine, China Agricultural University, Beijing 100193, China

**Keywords:** intestinal homeostasis, iron overload, ferroptosis, inflammation, intestinal diseases, therapy

## Abstract

Intestinal homeostasis is maintained through the interplay of the intestinal mucosa, local and systemic immune factors, and the microbial content of the gut. Iron is a trace mineral in most organisms, including humans, which is essential for growth, systemic metabolism and immune response. Paradoxically, excessive iron intake and/or high iron status can be detrimental to iron metabolism in the intestine and lead to iron overload and ferroptosis-programmed cell death mediated by iron-dependent lipid peroxidation within cell membranes, which contributes to several intestinal diseases. In this review, we comprehensively review recent findings on the impacts of iron overload and ferroptosis on intestinal mucosal homeostasis and inflammation and then present the progress of iron overload and ferroptosis-targeting therapy in intestinal diseases. Understanding the involved mechanisms can provide a new understanding of intestinal disease pathogenesis and facilitate advanced preventive and therapeutic strategies for intestinal dysfunction and diseases.

## 1. Introduction

The intestine represents the largest compartment of the immune system. It is continually exposed to antigens and immunomodulatory agents from the diet and commensal microbiota, and it is the port of entry for many clinically important pathogens [1]. The intestinal mucosa comprises a layer of polarized, columnar epithelial cells and a subepithelial region that contains the lamina propria, enteric nervous system, connective tissue, and muscular layers [2]. The epithelium includes intestinal stem cells, enterocytes, goblet cells, Paneth cells, tuff cells and enteroendocrine cells. These intestinal epithelial cells (IECs) provide a physical and biochemical barrier that segregates host tissue and commensal bacteria to maintain intestinal homeostasis [3]. Once the mucosal barrier is destroyed or the microbiota is imbalanced, excessive inflammatory responses will be activated, resulting in the destruction of the intestinal structure, translocation of pathogens, and progression of inflammation and metabolic diseases [4,5,6,7]. Therefore, the mucosal barrier plays a crucial role in maintaining intestinal homeostasis. Above the epithelial barrier is the mucus layer, which mainly contains microbiota, secretory IgA, mucins, antimicrobial peptides and glycocalyx. The lamina propria includes a diffuse lymphoid tissue made up of macrophages, dendritic cells, plasma cells and lymphocytes, and a structured lymphoid tissue made up of Peyer’s patches, which contain M cells, dendritic cells, and lymphocytes, which have important roles in immune response and inflammation in the intestine.

Iron is an essential trace element in the body, and most iron is stored in ferritin and incorporated into heme and [Fe-S] cluster proteins. Iron is involved in a wide variety of biological processes, such as oxygen transport, ATP generation, energy metabolism and DNA synthesis and repair [8]. Therefore, sufficient iron is required for cells. In mammalian cells, iron is distributed in mitochondria (approximately 16 μM), cytosol (approximately 6 μM), nuclei (approximately 7 μM) and lysosomes (approximately 16 μM). Although the appropriate amount of iron is indispensable for cells, the very small amount of free iron, which is called the cellular labile iron pool (LIP) and is composed of redox-active iron (Fe^2+^), can damage several cellular functions. Redox-active iron can form reactive oxygen species (ROS), which damages DNA, proteins and lipids [9,10]. These damaged molecules can trigger senescence or cell death, leading to various diseases. In the intestine, most iron absorption occurs in the duodenum and jejunum. Nevertheless, iron supplementation results in excess iron accumulating in the intestinal tract that can also catalyze the formation of ROS and cause intestinal damage, which increases intestinal susceptibility to the toxic effects of iron overload [11,12]. Numerous studies have shown that iron overload can cause iron deposition in the intestinal mucosa, which in turn damages intestinal mucosal homeostasis and increases inflammation and lipid peroxidation as well as cell death, such as ferroptosis, resulting in various intestinal diseases [12].

Ferroptosis is a novel type of cell death with distinct properties and recognizing functions involved in physical conditions and various diseases [13]. It is induced by severe lipid peroxidation depending on ROS generation and iron overload [14]. As a highly iron-dependent programmed cell death, ferroptosis manifests evident morphological, biochemical and genetic differences from other forms of regulated cell death, such as apoptosis, autophagy, necrosis and pyroptosis [15,16,17,18,19,20]. In multicellular organisms, ferroptosis is involved in a series of fundamental cellular activities, including modulation of cell proliferation and various immune functions. Accumulating researches have demonstrated that ferroptosis can participate in multiple human diseases, including metabolic disorders, aging, nonalcoholic steatohepatitis, liver cirrhosis, neurodegeneration, ischemia/reperfusion (I/R) injury, inflammatory bowel disease (IBD) and several types of cancers [21,22,23,24,25,26,27,28,29,30]. Dysfunction of ferroptosis has been considered as a critical factor in damage of the intestinal mucosal barrier, which may result in the impairment of antimicrobial peptide secretion, bacterial elimination, and modulating intestinal inflammation and homeostasis. Given this evidence, researchers are increasingly paying attention to the protective mechanisms of ferroptosis in intestinal homeostasis. Therefore, this review presents the first comprehensive summary of the current understanding of intestinal mucosal homeostasis and iron metabolism, then focuses on the mechanisms of iron overload and ferroptosis as well as their impacts on intestinal mucosal homeostasis and inflammation. Finally, the progress of intestinal disease therapy by targeting iron overload and ferroptosis is presented.

## 2. The Importance of Maintaining Intestinal Mucosal Homeostasis

**Figure 1:**
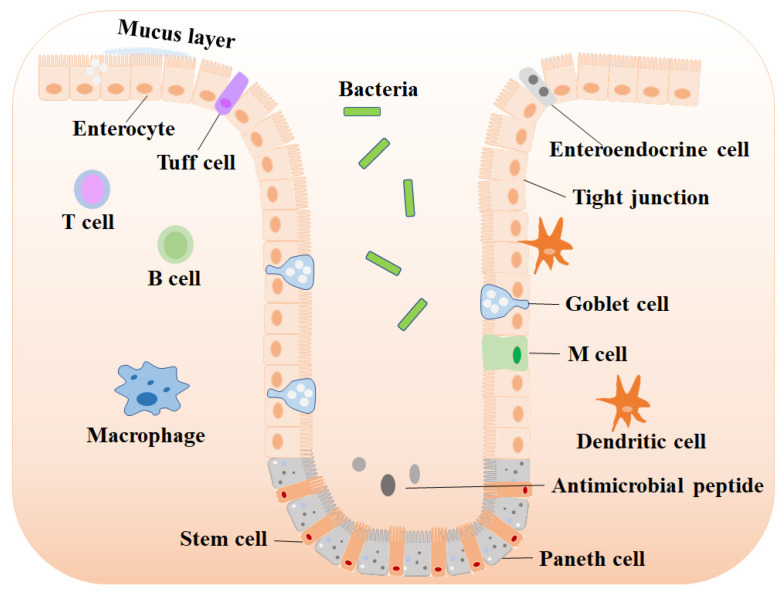
The structure of the intestinal mucosal barrier. The intestinal mucosal barrier consists of the epithelium layer, the mucus layer and immune cells. The intestinal epithelial cells include stem that locate at base invaginations and the differentiated cells (absorptive enterocytes, goblet cells, Paneth cells, tuff cells and enteroendocrine cells). Immune cells reside underneath the epithelium, which include dendritic cells, M cells, macrophages, B cells and T cells.

The mammalian intestine comprises the mucosal barrier, the microbiota and the host immune system (Figure 1). 

### 2.1. The Role of the Intestinal Barrier 

The mucosal barrier takes an important part in keeping intestinal homeostasis, which mainly consists of IECs that are linked by tight junctions (TJs) and their protective mucous layer [31]. This epithelium is constantly exposed to food and microbes and acts as the first line of defense against microbial invasion [32,33]. The different types of IECs are originally differentiated from intestinal stem cells (ISCs) that reside at base invaginations, termed crypts of Lieberkühn. The differentiated cells include the absorptive enterocytes and various types of secretory cells (antimicrobial-secreting Paneth cells, tuff cells, mucus-secreting goblet cells, and hormone-producing enteroendocrine cells [34]. Most epithelial cells move out of the crypt base to the mucosal surface, where they exert functions, undergo death, and are shed into the intestinal lumen [35]. The intestinal barrier integrity is essential for the absorption of nutrients and health in humans and animals, and dysfunction of the IECs has been associated with increased sensitivity or spontaneous activation of inflammatory responses [35].

#### 2.1.1. Intestinal Stem Cells (ISC)

Intestinal stem cells residing at the bottom of the crypt are essential for maintaining homeostasis of the intestinal epithelium. The intestinal epithelium renews every 2–5 days, which is totally fueled by intestinal stem cells. ISCs include two small intestinal crypt-cell populations, leucine-rich repeat-containing G protein-coupled receptor 5 (Lgr5)-expressing crypt base columnar cells (CBCs) that cycle rapidly and are present predominantly at the crypt base, and Bmi1-expressing cells that largely reside above the crypt base [36,37,38]. Lgr5+ ISCs maintain the homeostasis of the intestinal epithelium in the steady state, while these cells are susceptible to epithelial damage induced by chemicals, pathogens, or irradiation. During the regeneration process of the intestinal epithelium, ISCs constitutively replenish to asymmetrically divide into two daughter cells, one maintaining as a stem cell for self-renewal at the crypt and the other becoming a transient amplifying (TA) cell to undergo differentiation [39]. TA cells experience 4–5 divisions, move toward the crypt-villus junction and then differentiate into all the epithelial cell types [40]. In general, ISCs support the continuous and rapid regeneration of intestine epithelium. Noticeably, the rates of cell proliferation and death in the intestine should balance exactly and be under strict controls, otherwise, the epithelial integrity will be disturbed and cause disorders [7,41]. Therefore, the maintenance of ISC function is essential in keeping intestinal homeostasis.

#### 2.1.2. Absorptive Enterocytes

Enterocytes are most abundant and make up more than 80% of the epithelial cell layer. Major tasks of enterocytes are digestion and absorption of nutrients and drugs/pharmacological agents, barrier permeability to micro-organisms, toxins and antigens, and transcytotic crosstalk between the intestinal lumen and lamina propria cells with access to the circulation. Enterocytes of the intestinal barrier act as sensors for antigens from nutrients and the intestinal microbiota, which they deliver to the underlying immune system of the lamina propria, triggering an immune response [42]. The immune function of enterocytes is the presentation of luminal antigens endocytosed at the apical membrane, processed in the endosomal pathway, conjugated to MHC class II antigens within late endosomes and finally recognized at the basolateral membrane by T lymphocytes. In vertebrates, four different types of cell junctions occur between two adjacent enterocytes: tight junctions (TJs), adhere junctions (AJs), gap junctions (GJs) and desmosomes. Among these, TJs are the most critical and responsible for material transport, physical defense, and maintenance of barrier functions [43,44]. Defects in the TJ barrier result in increased intestinal permeability and contribute to the development of intestinal inflammation and IBD [45].

#### 2.1.3. Enteroendocrine Cells

Enteroendocrine cells are responsible for orchestrating metabolism, insulin secretion, food intake, and nutrient assimilation by releasing diverse hormones [46]. Gut hormones generate signals related to the rate of nutrient absorption, the composition of the luminal milieu and the integrity of the epithelial barrier. They are divided into many subtypes according to their specific products: I cells (cholecystokinin), K cells (glucose-dependent insulinotropic polypeptide), N cells (neurotensin), X cells (ghrelin), enterochromaffin cells (tachykinin 1 and tryptophan 5-hydroxylase 1), L cells (glucagon), and delta cells (somatostatin). They secrete multiple regulatory molecules that control physiological and homeostatic functions, particularly postprandial secretion and motility. They also play a pivotal role in the control of food intake, and emerging data add roles in mucosal immunity and repair. For example, enteroendocrine cells scattered in the intestinal tract play an important role in controlling nutrient uptake by recognizing nutrient-derived antigens in the lumen via microvilli structures and G protein-coupled receptors [47].

#### 2.1.4. Goblet Cells

Secretory goblet cells comprise approximately 16% of the small intestinal and 50% of the colonic epithelium. Goblet cells have a well-appreciated role in barrier maintenance through the secretion of multiple mucus, such as MUC1, MUC2, MUC5AC and MUC5B [48,49,50,51]. Among all mucins, MUC2 has been identified as a primary component to form an intact mucus layer [52]. In addition, GCs can secrete RELM-β along with mucins, which have direct bactericidal properties, including killing commensal bacteria and pathogens that penetrate into the mucus layer [53,54,55]. Other components found in the inner layer of the mucus, such as the zymogen granulae protein 16 (ZG16) aggregates bacteria, preventing adherence of the bacteria to the epithelium [56]. Goblet cells secrete mucus that forms the epithelial barrier against pathogen invasion and can present antigens to CD103+ DCs in the lamina propria [57,58]. While GCs have essential roles in maintaining homeostasis, it is worth noting that GCs can also contribute to disease pathogenesis. GC dysfunction has been associated with and contributes to multiple diseases, including IBD, cystic fibrosis, asthma, metabolic disorders and chronic obstructive pulmonary disease [59].

#### 2.1.5. Paneth Cells and Tuff Cells

A characteristic feature of small intestinal host defense is the presence of Paneth cells at the bottom of the crypts of Lieberkühn. The Paneth cell, first described by Austrian physiologist Joseph Paneth, is a prominent secretory cell type located at the crypt base in a relatively small number (2%) and adjacent to ISCs [60]. Paneth cells reside at the crypt bottom, expressing defensins and other antibiotic peptides (e.g., lysozyme) to keep the intestinal crypt lumen aseptic [61]. Except for bactericidal products, Paneth cells serve as multifunctional guardians of stem cells and can support essential niche factors for the maintenance of the adjacent stem cells, such as stimulating ISC differentiation, shaping the intestinal microbiota and augmenting ISC function [62,63]. Additionally, tuft cells can act as novel niche cells on Paneth cell ablation. Even if Paneth cells are ablated, intestinal homeostasis is not impaired due to tuft cells compensating for the loss of Paneth cells [39,64].

### 2.2. The Role of Intestinal Microbiota

A great number of investigations have shown that more than 100 trillion bacteria inhabit the lower gastrointestinal tract of humans, whose collective genome (“microbiome”) contains at least 100 times as many genes as our own genome [65]. Notably, the vast majority of these are nonpathogenic bacteria that are essential to human health [66]. The gut microbiota is influenced by factors, such as age, gender, diet and immune status and the interaction between the host and these microbes is largely symbiotic. These resident bacterial populations make a number of key contributions to host health, including: (1) enhancing nutrient digestion and absorption; (2) producing vital energy sources, such as short-chain fatty acids; (3) regulating metabolism and synthesizing important vitamins; (4) competing with pathogens and limiting their colonization; and (5) promoting the development and maturation of the immune system [67,68,69,70]. In return, resident micro-organisms benefit from association with their hosts by inhabiting a protected, nutrient-rich environment [71]. Thus, these host-microbial associations constitute a mutually beneficial symbiosis.

### 2.3. The Role of Intestinal Immune Cells

In the intestine, the immune system contains the lamina propria (LP) and gut-associated lymphoid tissues (GALT), such as mesenteric lymph nodes and Peyer’s patches (PP) [71]. The PP are important induction sites that contain all the immune-competent cells necessary to induce antigen-specific responses [1]. In the intestine, macrophages, M cells, dendritic cells (DCs), T cells, B cells, and natural killer cells are important components of the intestinal mucosal immune system. There is an intense communication between epithelial cells, immune cells and the microbiome that shapes specific immune responses to antigens, balancing tolerance and effector immune functions. Once this balance of the mucosal barrier is disturbed, either by alterations of the microbiota composition or by changes to host responses, dysbiosis and intestinal dysfunction will occur and even lead to numerous diseases, such as metabolic syndromes and IBD [68,72].

#### 2.3.1. Macrophages and M Cells

Macrophages are part of innate immunity and are key players in the maintenance of intestinal homeostasis. They belong to the group of mononuclear phagocytes that exert bactericidal functions and help to clear apoptotic cells. Moreover, they have essential roles in the maintenance of epithelial integrity, the development of intestinal inflammation, tissue remodeling during wound healing processes, and they are involved in intestinal tumor development. Macrophages are antigen-presenting cells and secrete immune-modulatory factors, such as chemokines and cytokines, which are necessary to activate other intestinal immune cells and therefore to shape immune responses in the gut. Generally, macrophages have been classified into two subgroups. It became evident that mononuclear phagocytes and especially macrophages in the intestine can play important roles in the development of IBD, which are characterized by increased expression of the pro-inflammatory markers TNFα, IL-1β, IL-6, and iNOS [73,74,75,76,77,78].

In addition, M cells are specialized epithelial cells of the follicle-associated intestinal epithelium. They act as an antigen sampling system and have a high capacity for transcytosis of a variety of micro-organisms and macromolecules into the subepithelial dome [79]. The primary role of M cells is the rapid uptake and presentation of particular antigens and micro-organisms to the immune cells to induce an effective immune response. Additionally, the low number of M cells in the intestine and the direct contact to immune cells can also prevent intestinal mucosal inflammation [79].

#### 2.3.2. T and B Cells

The intestine comprises the largest compartment of the immune system, with substantial amounts of organized lymphoid tissue and large populations of scattered innate and adaptive effector cells [1]. These cells play important roles in antimicrobial defense, and contribute to organ development, tissue protection and regeneration and mucosal homeostasis by maintaining the balance between antipathogen immunity and commensal tolerance [80,81,82]. Activated B cells differentiate and produce secretory immunoglobulin (Ig) A, which can help to control the growth of intestinal bacteria and prevent microbial adhesion to epithelial cells [83]. Conventional gut-resident T cells mainly include CD8+ T cells and CD4+ T cells; the latter are generally composed of T-helper 1 (Th1) cells, T-helper 2 (Th2) cells, T-helper 17 (Th17) cells, follicular helper T (Tfh) cells and regulatory T (Treg) subsets. Among them, Th1 cells are essential mediators in the eradication of intracellular pathogens, such as viruses and bacteria, by producing IFN-γ [84]. Th17 cells, which produce IL-17 and/or IL-22, have a critical role in host defense against fungi and maintenance of intestinal homeostasis [84].

#### 2.3.3. Dendritic Cells

DCs are a heterogeneous population of innate immune cells composed of classical and monocyte-derived DC, Langerhans cells, and plasmacytoid DCs. In the intestine, DCs are found in organized lymphoid tissues, such as the mesenteric lymph nodes and Peyer’s patches, as well as in the lamina propria [85]. These DCs have the responsibility of initiating immune responses against mucosal pathogens. Depending on environmental stimuli, they secrete various cytokines and cell mediators, mainly IL-10, TGF-β, IL-6, IL-4, IL-12, B-cell activating factor (BAFF) and a proliferation-inducing ligand (APRIL) and induce the differentiation of T and B cells. Among them, IL-10, TGF-β, IL-6, IL-4 and IL-12, induce the differentiation of T helper (Th) cell precursors (Th0) into effector Th cells (mainly Th1, Th2, Th17) or regulatory Th cells (Tregs, mainly Th3), while BAFF and APRIL induce the differentiation of B cells into IgA-producing plasma cells in a T cell-independent manner [86,87,88,89].

## 3. Intestinal Iron Metabolism 

**Figure 2:**
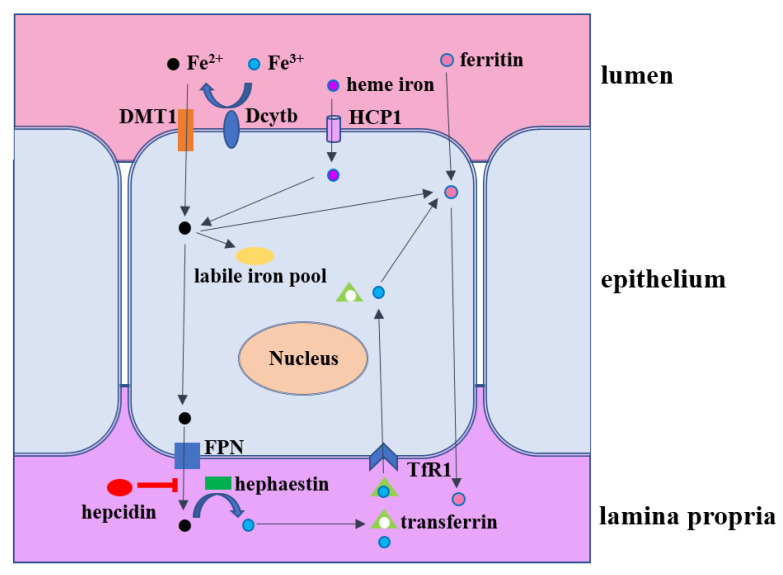
The intestinal iron metabolism. Heme iron is entirely absorbed by enterocytes through heme carrier protein 1 (HCP1). Divalent metal ion transporter (DMT1) can transport Fe^2+^ reduced by duodenal cytochrome b reductase (Dcytb). Iron passes through the basement membrane through ferroportin 1 (FPN1) or is stored by intracellular ferritin and the labile iron pool (LIP). Fe^2+^ is converted into Fe^3+^ by hephaestin (Hp) and then combined into transferrin into the serum. Transferrin receptor 1 (Tfr1) expressed in the intestinal epithelium is able to facilitate cellular iron acquisition by binding to and internalizing iron-loaded transferrin. FPN1 is controlled by hepcidin to induce internalization and the subsequent degradation of ferroportin to regulate iron output. Furthermore, ferritin has been found to be able to pass through intestinal epithelial cells independent of DMT1 channel or heme transporter PCFT/HCP1.

The mechanism that regulates intestinal iron absorption is complicated (Figure 2). Iron is absorbed by the mature enterocytes of the midupper villus and mainly in the small intestine. Commonly, two types of iron can be absorbed in the intestine that includes heme iron and non-heme iron [90]. Heme iron comes from hemoglobin and myoglobin in animal foods, which has high bioavailability and is entirely absorbed by enterocytes through heme carrier protein 1 (HCP1) [91,92]. Due to cost constraints, non-heme iron is a common iron supplement in feed. To move from the lumen of the intestine into the bloodstream, iron must cross both the apical brush-border membrane and the basolateral membrane of enterocytes. For non-heme iron, it traverses the brush-border membrane via divalent metal ion transporter (DMT1), which requires ferrous iron (Fe^2+^) as a substrate. As most dietary iron is in the ferric (Fe^3+^) form, this form of iron needs to be reduced by duodenal cytochrome b reductase (Dcytb) before it can be absorbed [93]. Two cytoplasmic proteins of iron regulatory proteins 1 and 2 (IRP1 and IRP2) have a central role in the regulation of cellular iron homeostasis by a post-transcriptional feedback mechanism, which can regulate iron metabolism-related proteins (DMT1, ferroportin, ferritin etc.) and maintains the LIP at an appropriate level [94]. If iron in the enterocyte is not immediately required by the body, it becomes sequestered in the cell within the iron storage protein ferritin only to be lost from the body once the enterocyte is sloughed at the end of its several day life span [10]. If iron is required, it can be exported rapidly across the enterocyte basolateral membrane via ferroportin 1 (FPN1) [10]. Fe^2+^ is converted into Fe^3+^ by hephaestin (Hp) and then combined into transferrin into the serum, which is transported throughout the body [95]. Transferrin receptor 1 (Tfr1), expressed in the intestinal epithelium, is able to facilitate the cellular iron acquisition by binding to and internalizing iron-loaded transferrin [96]. FPN1 is controlled by hepcidin to induce internalization and the subsequent degradation of ferroportin to regulate iron output [97]. The post-translational regulation of ferroportin by hepcidin may thus complete a homeostatic loop: Iron regulates the secretion of hepcidin, which in turn controls the concentration of ferroportin on the cell surface [97]. Recently, ferritin has been found to be able to pass through intestinal epithelial cells and accumulate in the blood, and its transport process is independent of DMT1 channel or heme transporter PCFT/HCP1 [98]. In terms of heme iron, very little is known definitively about the absorption of heme iron. It is presumed to bind to the enterocyte brush border intact and then might be endocytosed. Once within the enterocyte, iron is released from heme through the action of heme oxygenases and subsequently exported from the cells via FPN1, which is similar to the pathway of non-heme iron [10].

## 4. Microbial Iron Absorption Mechanism

In addition to the absorption and utilization of iron in human intestines, the intestinal microbiota also require iron for life activities. As an essential element, iron is also extensively required across the domain of bacteria by functioning as a co-factor in iron-containing proteins for redox reaction, metabolic pathways, and electron transport chain mechanisms [99]. Iron is critical for the replication and survival of almost all bacteria, with a few exceptions, which acquires alternative metabolic solutions from evolution. As with humans, these gut residents have evolved a number of mechanisms for obtaining iron from their human hosts for survival and proliferation [100]. Micro-organisms have a high-affinity iron absorption mechanism, which can be divided into three categories: iron carrier-based systems, heme acquisition systems and transferrin/lactoferrin receptors [101]. Siderophores are small, high-affinity iron-chelating compounds that are secreted by bacteria, and they are the most prevalent strategies of aerobic and facultative anaerobic bacteria families, such as *Enterobacteriaceae*, *Streptomycetaceae*, and *Bacillaceae*, in order to scavenge inorganic iron from the environment [102]. They are vastly produced by bacteria under low iron stress, due to their high ferric ion-specific chelating capacities. There is no shared protein structure of siderophores due to the ability of the gut bacterial species to produce iron-siderophore complexes with specific transporters. Several Gram-negative bacteria, such as *Neisseriae*, require heme-associated iron to support bacterial growth. As in the case of intact iron-loaded siderophores, the entire heme is taken up via outer membrane receptors and transported into the bacterial periplasm, whereas heme-containing proteins are unloaded at the cell surface and the heme is transported to the periplasm. Once in the periplasm, the heme is then transported by specific ABC protein-dependent periplasmic permeases through the inner membrane [103,104]. In addition, transferrin/lactoferrin receptors play a role in transporting iron on the surface of bacteria. Lactoferrin is a critical mediator of both host immune response and antimicrobial activity in response to Streptococcal infections, which has the strong ability to bind iron with high affinity and sequester this important nutrient from an invading pathogen [105]. Iron acquisition and storage control are mediated in most bacteria by the global ferric uptake regulator (Fur) [106]. Fur is a transcription factor that utilizes Fe^2+^ as a corepressor and represses siderophore synthesis in pathogens. Fur, directly or indirectly, controls the expression of enzymes that protect against ROS damage. Thus, the challenges of iron homeostasis and defense against ROS are addressed via Fur. Although the role of Fur as a repressor is well-documented, emerging evidence demonstrates that Fur can function as an activator through three distinct mechanisms: (1) indirectly via small RNAs; (2) binding at cis regulatory elements that enhance recruitment of the RNA polymerase holoenzyme (RNAP) and (3) functioning as an anti-repressor by removing or blocking DNA binding of a repressor of transcription [106]. In addition, Fur also regulates many virulence factors of pathogenic bacteria by mediating key pathogen responses, such as invasion of eukaryotic cells, toxin production, motility, quorum sensing, stress resistance or biofilm formation. Therefore, Fur plays a major role in iron homeostasis and pathogenicity of bacteria. 

## 5. The Impacts of Iron Overload on Intestinal Homeostasis

**Figure 3:**
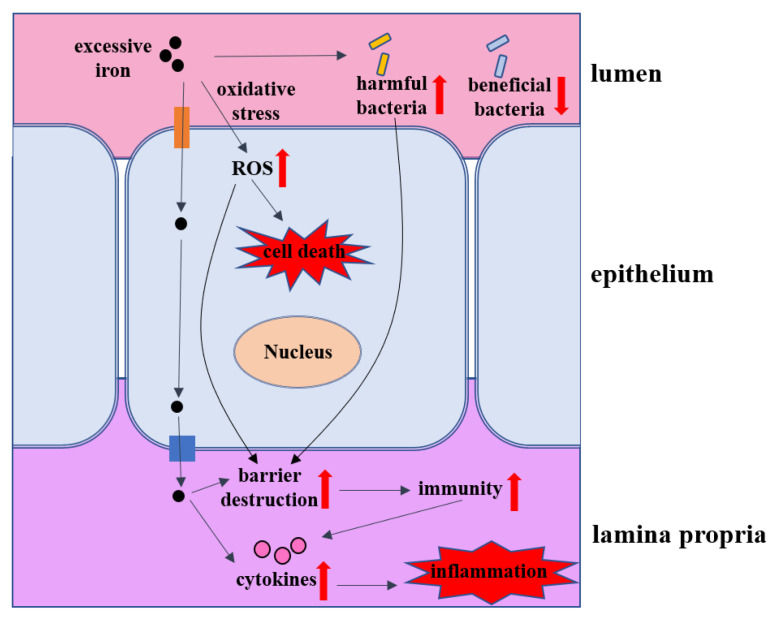
The impacts of iron overload on intestinal homeostasis. The impacts of iron overload on intestine mainly focus on the changes of the intestinal barrier, intestinal immunity, inflammation, intestinal microbes, transport channels and cell death. Excessive iron can damage the intestinal barrier by iron-induced ROS and exacerbate the inflammatory response. Iron overload can cause the disturbances in the intestinal microbiota, which reduces the number of probiotics, while increasing the number of pathogenic bacteria. In addition, iron-induced ROS can result in intestinal cell death, including ferroptosis.

Various cell types in the intestine, including epithelial cells and macrophages, can produce iron metabolism-related proteins to regulate iron homeostasis and protect intestine tissue from oxidative stress. Iron metabolism disorders are closely related to intestine tissue damage in patients, that is, too much iron can generate ROS and cytotoxicity through the Fenton reaction and consequently cause oxidative damage to cells and tissues that can lead to tissue fibrosis and organ dysfunction long term [107]. Excessive absorption of dietary iron into the plasma is associated with the presence of a labile iron known as non-transferrin-bound iron (NTBI), which plays a major role in tissue iron overload [108]. Most iron absorption occurs in the duodenum and jejunum, increasing their susceptibility to the toxic effects of iron overload [109]. As shown in Figure 3, we introduce the effects of iron overload on the intestine from the perspective of the intestinal barrier, intestinal inflammation, intestinal microbes, transport channels and cell death.

### 5.1. Intestinal Barrier

High-dose iron-induced oxidative stress destroys epithelial tight junctions and intestinal barrier permeability. Studies have shown that iron overload can cause iron deposition in the intestinal mucosa, which in turn aggravates the gastrointestinal mucosal injury, leading to increased inflammation and lipid peroxidation [110]. As a result, patients can suffer from gastrointestinal complications, such as diarrhea, vomiting, and digestive tract injury, after excessive iron intake [111]. Ferric citrate (FC) is an iron-containing phosphate binder used as a food additive for iron supplementation. Luo et al. explore the potential effect of FC on intestinal epithelial function and find that long-term oral administration of FC causes iron overload in the intestine tissue and serum in mice [12]. The villus height, the ratio of villus height to crypt depth, and the number of intraepithelial lymphocytes and goblet cells in the jejunum are all decreased. Other investigators demonstrate that FC can weaken the intestinal epithelial tight junction [112]. Moreover, iron overload increases serum D-lactate acid (D-LA) levels and decreases tight junction proteins (claudin-1, occludin, and ZO-1), MUC-2, and TFF3 [12]. Thus, iron overload resulting from the chronic oral administration of FC impairs the intestinal immunity and barrier in mice. In addition, oral administration of liquid iron preparation containing excess iron impairs intestinal barrier function and causes inflammation as well as oxidative stress in rats, which decreases the expression of ZO-1 and occludin (representatives of tight junction proteins) in the colon [108]. The increased supplementation of dietary iron in the diet of young pigs could also increase intestinal permeability, ion transport and inflammation, suggesting that adequate but not over-supplementation of dietary iron in pigs is required to maintain intestinal barrier health and function [110]. D-LA, diamine oxidase (DAO) and fatty acid-binding protein 2 (FABP2) provide a barrier function by intact intestinal mucosa and are indices of high intestinal permeability the levels of which are increased in rats by long-term intake of iron (1000 mg/kg, 12 weeks) in diet [113,114]. 

### 5.2. Intestinal Inflammation

High iron content in the diet affects the composition of the microbiome and stimulates the immune response of the lamina propria, as iron easily accumulates in the colon [90]. Intestinal epithelial cells are destroyed by iron-induced ROS, which leads to an incomplete intestinal mechanical barrier and exacerbates the inflammatory response. For example, iron overload caused by long-term oral administration of FC also upregulates the pro-inflammatory cytokines (IL-1β, IL-2, IL-6, TNF-ɑ), while downregulating the anti-inflammatory cytokines (IL-4, IL-10) and sIgA in the intestine of mice [12]. Duodenal mucosal tumor necrosis factor α (TNFα), interleukin (IL) -1β and IL-6 relative gene expression are upregulated by 36%, 28%, and 45%, respectively, in pigs with high dietary iron (520 mg Fe/kg) [110]. 

### 5.3. Intestinal Microbes

The gut microbiota plays a key role in the nutrition and health of its host [115]. However, gut microbiota can rapidly respond to altered diets and dietary metals [116]. Excessive iron can destroy the balance of beneficial bacteria and harmful bacteria, which is helpful for the reproduction of harmful bacteria. For example, lower populations of *Bifidobacterium* and *Lactobacillus* spp. and higher populations of coliforms in the feces of pigs fed Fe-supplemented diets when compared with normal feces [117]. Under high concentrations of dietary iron, the number of total anaerobes in the colons of mice are also decreased [118]. Provision of iron-containing micronutrient powder (MNP) to weaning infants adversely affects the gut microbiome, increasing pathogen abundance and causing intestinal inflammation [119]. In children received iron-fortified food, there is a significant increase in the number of enterobacteria and a decrease in lactobacilli in the iron group along with increased gut inflammation [120]. High doses of ferrous sulfate reduce the number of probiotics, such as *Lactobacillus* and *bifidobacteria*, while increasing the number of pathogenic bacteria, such as *Escherichia coli* and *Salmonella typhimurium* [121]. Once pathogenic bacteria dominate, the intestinal inflammation may be worsened. In addition, oral administration of liquid iron preparation containing excess iron induces intestine injury and alters the gut microbiota in rats, with increased *Defluviitaleaceae*, *Ruminococcaceae*, and *Coprococcus* and reduced *Lachnospiraceae* and *Allobaculum*, which could mediate the detrimental effects of excess iron on gut health [108]. Not surprisingly, iron can promote the replication and virulence of gut enteric pathogens, including *Salmonella*, *Shigella*, and *Campylobacter* [100]. For example, oral iron supplementation induces pathogenic overgrowth of *Salmonella* and other enteric pathogens at the intestinal epithelial interface [122]. So far, dietary recommendations are the basis for preventing disturbances in the intestinal microbiota of humans and animals. Considering the harm of iron overload to intestinal health, it is particularly crucial to supply or limit the intake of iron scientifically. 

### 5.4. Intestinal Transport Proteins

High-dose iron in the diet can also affect the iron-related proteins in the intestine. For example, the expressions of FPN1 and DMT1 proteins were up-regulated in rats after co-treatment of ethanol plus iron compared to the control group [114]. Hepcidin regulates the degradation of intestinal ferroportin, and its mRNA abundance in the Fe-overload group (900 mg Fe) was seven times higher than that in the regular group, thus reducing the intestinal iron transport into the circulation system [123]. Studies in hereditary hemochromatosis (HH) patients have shown that despite the iron overloading in parenchymal organs, dietary iron absorption is relatively high, and that DMT1 and iron regulated-transporter-1 (Ireg1, also known as ferroportin or metal transporter protein-1, MTP1) in the intestines are expressed in inappropriately high levels with respect to body iron status [124,125]. In contrast, F Canonne-Hergaux et al. illustrate that no increase is seen in DMT1 expression in mice with iron overload resembling human hemochromatosis, and it seems that changes in DMT1 levels might not be primarily responsible for iron loading in hemochromatosis [126]. Therefore, the molecular mechanism of the relationship between DMT1 and iron overload needs to be further explored in the future. 

### 5.5. Cell Death

Excessive iron in the diet usually produces ROS in the intestine through the Fenton and Haber–Weiss reactions. ROS accumulation is the trigger for a wide variety of cellular stress responses, including DNA damage responses and other signal transduction-related responses [127]. For instance, it is well known that ROS mediated DNA damage activates the ATM/ATR-p53 pathway, resulting in cellular senescence or cell death [128]. Given that iron plays an important role in producing ROS, iron may well contribute to ROS-mediated senescence or cell death pathways, including apoptosis, necrosis and pyroptosis. ROS accumulation activates the apoptosis signal-regulating kinase 1 (ASK1)-p38/c-Jun N-terminal kinase (JNK) pathway, leading to apoptosis [129]. Additionally, ROS can oxidize cardiolipin and induce mitochondrial outer membrane permeabilization (MOMP) and cytochrome c release. Then, caspase-9 (CASP9) and subsequently caspase-3 (CASP3) are activated and induce apoptosis (intrinsic apoptotic pathway) [130]. In male Sprague-Dawley rats, oral administration of liquid iron preparation containing excess iron also increases apoptosis in duodenal enterocytes [108]. Necroptosis is a type of programmed necrosis that is dependent on the activation of receptor-interacting serine-threonine kinase 1/3 (RIPK1/3) and mixed-lineage kinase domain-like (MLKL). ROS accumulation can cause the formation of necrosomes containing RIPK1/3, which are activated by auto- or transphosphorylation. Activated RIPK3 phosphorylates and promotes MLKL oligomerization, then activated MLKL can form a supramolecular protein complex at the plasma membrane and ultimately execute necroptosis [131]. In terms of pyroptosis, iron-activated ROS signaling can cause the oxidation and oligomerization of the mitochondrial outer membrane protein Tom20. Then, Bax is recruited to mitochondria by Tom20 and induces cytochrome c release and caspase-3 activation. This caspase activation triggers cleavage of gasdermin (GSDME), whose high expression levels are critical for the switch from apoptosis to pyroptosis [132]. Recently, researchers have paid attention to the relationship between iron and a novel programmed cell death named ferroptosis. As an iron-dependent process, iron overload is the driving factor of ferroptosis, which aggravates intestinal injury. For example, one study has reported that non-heme iron (such as ferrous sulfate and ferric citrate) is likely to cause intestinal cell ferroptosis [133]. In other words, the cells appear to be overloaded with iron due to ferroptosis and the disease becomes increasingly worse. Thus, there is no doubt that ferroptosis plays a crucial role in the intestine diseases, which are specifically summarized as follows. 

## 6. Ferroptosis Machinery and Its Role in Intestinal Diseases

The term ‘ferroptosis’ was coined in 2012 to describe a newly-identified mechanism of programmed cell death mediated by iron and distinct from other known forms of programmed cell death, such as apoptosis [134]. Morphologically, cells undergoing ferroptosis have a typical necrotic morphology, along with small dysmorphic mitochondria with decreased crista, a condensed membrane, a ruptured outer membrane and no hallmarks of apoptosis [19,135]. A number of publications have since confirmed the existence of ferroptosis and characterized its impacts on both health and disease [136]. 

### 6.1. Major Metabolic Mechanisms of Ferroptosis 

**Figure 4:**
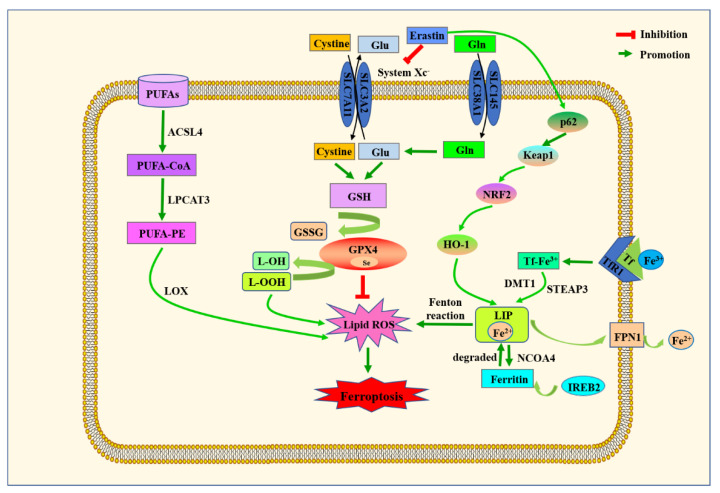
Mechanisms of ferroptosis. Ferroptosis is characterized by iron accumulation, excessive ROS production and overwhelming lipid peroxidation. This figure shows the regulatory pathways of ferroptosis, which are roughly divided into three categories: amino-acid/GSH, lipid and iron pathways.

Ferroptosis is a form of cell death that is regulated by multiple genes and involves multiple metabolic processes, such as amino acid metabolism, lipid peroxidation and iron metabolism. The mechanism is very complex, as is shown in Figure 4, and it will be better explained in the following aspects.

#### 6.1.1. Amino acid Metabolism

The first description of the mechanism of ferroptosis involved the action of erastin, named for eradicator of RAS and small T antigen-expressing cells, a small molecule that inhibits the cystine/glutamate antiporter system and, thereby, limits the intracellular availability of cystine required for glutathione (GSH) synthesis. Cystine ultimately generates GSH through a series of enzymatic reactions, and GSH is the essential substrate for glutathione peroxide enzyme 4 (GPX4) to degrade phospholipid hydroperoxide (PLOOH) [137]. GPX4 is at the intersection of GSH metabolism and lipid peroxidation, both of which are related to ferroptosis. The depletion of GSH and inactivation of glutathione peroxidase 4 (GPX4), a phospholipid hydroperoxidase that protects cells against lipid peroxidation, results in membrane lipid peroxidation that builds up to toxic levels and kills the cell [17,19].

#### 6.1.2. Lipid Metabolism

The most prominent feature of ferroptosis is plasma membrane damage caused by the production of iron-dependent lipid peroxides (lipid ROS) [138]. ROS includes products of oxygen reduction, such as O^2−^, H_2_O_2_ and -OH. Oxygen homeostasis is crucial to normal cellular functions, and the abnormal accumulation of ROS is harmful to the body [139]. During ferroptosis, the reduction reaction mediated by GPX4 and ferroptosis suppressor protein 1 (FSP1, formerly known as mitochondrial apoptosis inducing factor 2, AIFM2) is inhibited, and the oxidation reaction catalyzed by Fe^2+^ and a series of iron-dependent enzymes (mainly LOX) is enhanced, inducing the accumulation of polyunsaturated fatty acids (PUFAs) [138]. Then, lipid peroxidation driven by PUFAs increases the permeability of the cell membrane and makes the cell more sensitive to oxidation, which eventually leads to ferroptosis [140]. The inhibition of lipid peroxidation and the consumption of PUFAs can inhibit ferroptosis [141].

#### 6.1.3. Iron Metabolism

Iron overload is one of the key events in ferroptosis. Iron is necessary for the accumulation of lipid peroxides, and iron ingestion, storage and transport all affect ferroptosis [141]. Iron homeostasis is regulated by a series of iron regulatory proteins (IRPs). Extracellular iron enters the cell through transferrin (Tf) and its receptors and then Fe^2+^ can produce lipid peroxides via the Fenton reaction or the iron-containing enzyme lipoxygenase (LOX) [138]. Most intracellular Fe2^+^ is stored in ferritin (FT) and so there is very little free Fe^2+^ [128]. The degradation of FT increases the level of intracellular Fe^2+^, enhances lipid peroxidation, and induces ferroptosis. This process is related to autophagy and is regulated by nuclear receptor coactivator 4 (NCOA4) [138]. Iron response element binding protein 2 (IREB2) and other proteins related to iron metabolism (HSPB1, CISD1, etc.) can also increase the sensitivity of cells to ferroptosis [19,141,142].

### 6.2. The Role of Ferroptosis in Intestinal Diseases

Accumulating investigations have proved the roles of ferroptosis in a range of intestinal diseases. Interestingly, ferroptosis appears to be a double-edged sword, which has positive functions in cancer, while showing negative functions in other intestinal diseases. Here, we summarize the interaction between ferroptosis and intestinal diseases to facilitate the clinical therapy.

#### 6.2.1. Inflammatory Bowel Disease (IBD)

IBD, including ulcerative colitis (UC) and Crohn’s disease (CD), are chronic inflammatory disorders of the gastrointestinal tract. Previous study has systematically summarized the worldwide incidence and prevalence of IBD in the 21st century (Ng et al., 2017). Their statistical report points out that the highest prevalence values of IBD are in Europe and North America; in some industrialized countries, such as China, the incidence rate is also accelerating, making IBD a global disease. IBD is associated with morbidity, mortality, and high costs of treatment. In patients with IBD, ROS is increased and GPX4 is decreased, and ferroptosis-related genes are also found to have changed [63,143]. In addition, Wang et al. have reported that ferroptosis is induced in mice with colitis, as evidenced by iron overload, GSH depletion, ROS and MDA production, accompanied by decreased expression of SOD and GPX4 [144]. Furthermore, similar effects of CUR on ferroptosis are observed in IEC-6 cells under the combined treatment of H_2_O_2_ and iron chloride hexahydrate [144]. Similarly, the expression of COX2 and ACSL4 is increased, while the level of GPX4 and FTH1 is deceased in 3% DSS-induced UC in mice. In terms of UC, a previous study has reported that IECs in CD exhibit impaired GPX4 activity and signs of lipid peroxidation, and PUFAs can trigger a cytokine response of IECs which is restricted by GPX4 [145,146]. 

There are two regulatory pathways that involves in ferroptosis in UC. Xu et al. have demonstrated that ferroptosis induced in the IECs from UC patients and mice with colitis is regulated by endoplasmic reticulum (ER) stress signaling [143]. Ferroptosis contributes to UC via ER stress mediated IEC cell death and NF-κBp65 phosphorylation can interact with eIF2α and suppress ferroptosis. Nrf2, a transcription factor, plays a key role in antioxidation processes [147]. Accumulating evidences have strongly indicated that Nrf2 is in charge of the maintenance of cellular homeostasis under stress and participates in ferroptosis [148]. Moreover, excessive activation of Nrf2/HO-1 induces ferroptosis through disturbing the balance of iron ion metabolism [149,150]. Chen et al. have validated that ferroptosis may play a vital role in UC associated with the Nrf2/HO-1 signaling pathway in which the expression of Nrf2 and HO-1 are dramatically increased in DSS-induced UC mice [147]. Above all, ferroptosis can aggravate IBD, so taking this machinery into consideration can provide a new perspective on drug design and make better use of the already-approved drugs in IBD treatment. 

#### 6.2.2. Colorectal Cancer (CRC)

Another fatal intestinal disease is colorectal cancer (CRC), which is the third most commonly diagnosed malignancy and the fourth leading cause of cancer-related death globally [151]. The worldwide burden of CRC is expected to rise by 60% to exceed 2.2 million new cases and 1.1 million deaths by 2030 [152]. Therefore, it is imperative to understand the pathogenesis of CRC and seek more effective therapeutic strategies. Substantial evidence demonstrates that ferroptosis plays dual and vital roles in tumorigenesis and CRC development. For example, erastin and cisplatin are found to induce ferroptosis in CRC cells, which can be inhibited by p53 [153,154]. RSL3 inhibits GPX4 activity and induces ferroptosis on three different CRC cell lines in vitro in a dose- and time-dependent manner due to increased ROS and an increase in the cellular labile iron pool, indicating that induction of ferroptosis contributes to RSL3-induced cell death in CRC cells and ferroptosis may be a pervasive and dynamic form of cell death for cancer treatment [155]. Guo et al. verify that the chemotherapeutic drug of cisplatin is an inducer for ferroptosis in human colorectal cancer cell line HCT116, and combination therapy of cisplatin and erastin shows a significant synergistic effect on anti-tumor activity [156]. 

Except that RSL3 inhibits GPX4 activity, several researchers have also implicated some molecules in ferroptosis in CRC are mediated by GPX4. For example, resibufogenin is able to inhibit colorectal cancer cell growth and tumorigenesis through triggering ferroptosis and ROS production mediated by GPX4 inactivation [157]. Ferroptosis can be triggered by iron-dependent lipid peroxidation after inactivation of the cystine/glutamate antiporter system xc^–^, which is composed of solute carrier family 7 membrane 11 (SLC7A11) and solute carrier family 3 membrane 2 (SLC3A2). Zhang et al. have firstly validated that the benzopyran derivative 2-imino-6-methoxy-2H-chromene-3-carbothioamide (IMCA) induces ferroptosis mediated by SLC7A11 through the AMPK/mTOR pathway in CRC [158]. Colorectal CSCs exhibit a remarkably lower level of ROS, a higher level of cysteine, glutathione and SLC7A11 compared to colorectal cancer cells; knockout of SLC7A11 can increase the ROS level and reduce the levels of cysteine and glutathione, subsequently attenuating the viability of colorectal CSCs [159]. Therefore, colorectal CSCs are more sensitive to ferroptosis, which could be targeted to attenuate colorectal cancer progression and chemoresistance. 

The role of heme oxygenase-1 (HO-1) in cancer biology is poorly understood. In fact, HO-1 has been described as survival molecule because of its anti-apoptotic and pro-angiogenic effects in several cancer types and its modulation can be induced by several natural compounds, such as polyphenols and terpenoids [160]. The HO-1 effect in cancer cells is not yet clear, but it is widely documented that HO-1 over-expression confers resistance to chemotherapy and radiation therapy due to its reaction products, such as CO or biliverdin/bilirubin [161]. In spite of its implication in tumor initiation, angiogenesis, and metastasis, excessively increased expression of HO-1 in tumor cells may lead to ferroptosis [162,163,164,165]. In fact, a lot of evidence shows that HO-1 induces ferroptosis through an increase of ROS production mediated by iron accumulation and accompanied by augmentation lipid peroxidation and glutathione depletion [149,166]. Malfa et al. have shown that *Betula etnensis* Raf. (Betulaceae) extract promotes ferroptosis in CRC cells by inducing heme oxygenase-1 (HO-1) expression [167]. ACSL-4 is an isozyme in the long-chain fatty-acid-coenzyme A ligase family and involves in the conversion of free long-chain fatty acids into fatty acyl-CoA esters, and thus plays a key role in lipid biosynthesis and fatty acid degradation. Recently, Doll and colleagues suggest that ACSL4 is critical for the induction of ferroptosis, and can predict sensitivity to ferroptosis by modulating phospholipids, specifically phosphatidylethanolamine [168]. Park et al. also demonstrate that bromelain effectively causes ferroptotic cell death in Kras mutant CRC cells compared to in Kras wildtype CRC cells by modulating ACSL-4 levels [169]. Hence, ferroptosis has a great potential to become a new approach in anti-tumor therapies and makes up for some classic drugs, which opens up a new way for their utility in the clinic.

#### 6.2.3. Intestinal Ischemia/Reperfusion (I/R) Injury

Intestinal (I/R) injury is a grave surgical event with high morbidity and mortality. Various pathological mechanisms can result in the occurrence of intestinal I/R injury, such as intestinal obstruction, severe trauma, intestinal transplantation, intestinal torsion and thrombosis [170]. Additionally, without appropriate diagnosis or treatment, the risk of increased mortality and morbidity becomes exponentially high [171]. There is a tight relationship between intestinal I/R injury and various types of cell death, including apoptosis, pyroptosis, and autophagy [172,173,174]. Nowadays, ferroptosis has also been found to have important roles in this disease. Following I/R injury, ROS is increased and GSH is declined as well as lipid peroxidation occurs [175,176]. Under ischemic conditions, the expressions of ACSL4 and iron are elevated, but GPX4 and GSH are decreased in the intestine, which can facilitate the occurrence of ferroptosis [23]. Liproxstatin-1, a potent and specific ferroptosis inhibitor, can induce GPX4 expression and ameliorate I/R-induced intestinal injury by inhibiting ferroptosis [23]. Inhibition of ischemia-induced ACSL4 is able to protect against ferroptosis following intestinal I/R. Furthermore, Sp1 is verified to regulate ACSL4 transcription in Caco-2 cells by binding to the ACSL4 promoter region [23]. 

In addition to the intestinal destruction, I/R can also cause downstream damage of other organs, such as the lung damage, and intestinal I/R-induced acute lung injury can enhance ferroptosis in vivo [177]. Erastin treatment of I/R mice significantly increases the percentage of MDA, Fe^2+^ levels and protein/mRNA levels of TF and significantly decreases the percentage of GSH and protein/mRNA levels of FTH1 and GPX4 [177]. p53, a tumor suppressor, is stimulated by apoptosis stimulating protein of p53 and consequently induces apoptosis and ferroptosis in response to DNA damage [178]. Interestingly, an inhibitor of apoptosis-stimulating protein of p53 (iASPP) inhibits p53-induced apoptosis and facilitates tumor growth, and the overexpression of iASPP induces chemoresistance in human cancer cells. Li et al. suggest that iASPP can inhibit ferroptosis and alleviate intestinal ischemia/reperfusion-induced acute lung injury via Nrf2 signaling [177]. Therefore, inhibition of ferroptosis is a potentially therapeutic method to protect humans against intestinal I/R, which is worthy of further investigation in the future. 

## 7. The Progress of Iron Overload and Ferroptosis-Targeting Therapeutic Strategies in Intestinal Diseases

**Figure 5:**
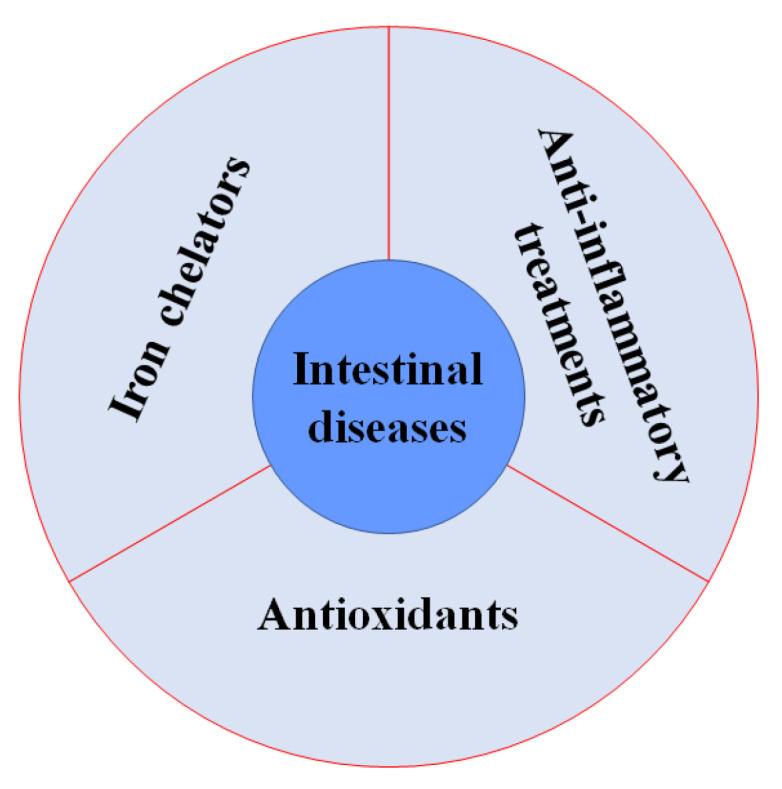
Treatments for intestinal diseases. Therapeutic targets for intestinal diseases associated with iron overload and ferroptosis, including iron chelators, antioxidants and anti-inflammatory treatments.

Disorders of iron homeostasis, iron overload, the depletion of GSH and oxidative stress are the key points leading to ferroptosis and can be considered as targets for the treatment of intestinal diseases. Notably, due to that, ferroptosis has a positive role in CRC via suppressing tumor growth and reducing the development of anticancer drug resistance; ferroptosis inducers can be used as anti-tumor therapies. As that a variety of diseases are caused by iron overload and ferroptosis, we mainly focus on the therapeutic strategies in several intestinal diseases by suppressing iron overload and ferroptosis in this study as shown in Figure 5. 

### 7.1. Iron Chelators

Iron chelators (deferoxamine, deferiprone, and deferasirox), especially deferoxamine (DFO), have been approved by the Food and Drug Administration (FDA) for the treatment of iron overload in patients [179,180]. In various animal models of infection, DFO has immunomodulatory effects to resist pathogens, such as bacteria, viruses, and fungi, in addition to chelating iron [181]. Particularly, iron chelators are able to decrease inflammation through the NF-kB signaling pathway [182,183]. In human IECs, DFO triggers inflammatory signals, including the production of CXC chemokine IL-8 by activating ERK1/2 and p38 kinase pathways [184]. In addition to some bacterial products known to induce host adaptive immune responses, direct chelation of host iron by infected bacteria may also contribute to the initiation of host adaptive immunity in the intestinal mucosa [185]. Moreover, administration of iron chelator can also decrease ROS levels in UC patients and diminish clinical signs [186]. In summary, iron chelators may also be effective in treating intestinal diseases, and the mechanism may be associated with the suppression of ferroptosis.

### 7.2. Antioxidants

Measures to reduce oxidative stress caused by iron overload are also worthy of attention, which can be decreased by antioxidants through inhibiting the formation of free radicals. Therefore, several therapeutic methods with antioxidant effects have been considered to be effective for intestinal diseases [187]. Due to the characteristics of economy, slight side effects and great therapeutic effect, antioxidants have become popular among humans suffering from intestinal diseases [188]. For example, COX-2 inhibitors and telmisartan (TLM) can inhibit ROS production and inflammation as well as increasing expressions of GSH and GPX in IBD patients [189,190]. Melatonin is a derivative of the essential amino acid tryptophan, which has various functions, including sleep promotion, blood pressure regulation, mitochondrial maintenance, immune modulation and as an antioxidant as well as exhibiting antiviral actions. As an antioxidant, it has a great protective effect against IBD that can act on the intestinal mucosal barrier [191]. Additionally, natural plants and herbal medicines also have substances with antioxidant effects to facilitate medications against intestinal diseases. Curculigoside (CUR) has been reported to relieve oxidative stress and intestinal damage by iron overload and ferroptosis in mice with UC through the induction of GPX4 [144]. Functional foods, such as camel’s milk, are also considered as useful antioxidants for IBD patients [192]. So far, it will still be required to validate the optimal dosage and route of medication for these potential drugs.

### 7.3. Anti-Inflammatory Treatments 

Inhibiting inflammation is an important treatment strategy for intestinal diseases. Combined inhibition of ferroptosis and inflammation has been reported to treat a variety of diseases, such as neurodegenerative diseases, stroke, myocardial infarction, pancreatitis and acute lung injury [142,193,194,195]. In terms of intestinal diseases, ferroptosis inhibitors have also been reported to be able to reduce inflammatory cytokines to treat diseases [182,183]. The molecular mechanism of ferroptosis and inflammation and ways to modulate inflammation by controlling ferroptosis are also required to be validated in the future to facilitate the novel strategies for clinical therapy of intestinal diseases.

## 8. Concluding Remarks

Iron plays a dominant role in the maintenance of IECs, the intestinal mucosal barrier and homeostasis. However, iron overload and induction of ferroptosis can show negative effects on intestinal mucosal homeostasis and inflammation, such as dysfunction of the epithelial barrier, increasing pathogen intracellular replication and proinflammatory cytokine production and also having a paradoxical role in some intestinal diseases. Current studies on the impact of iron overload and ferroptosis on intestinal mucosal homeostasis and inflammation provide advances in our comprehension of intestinal pathogenesis that may hopefully lead to novel and more effective therapeutic strategies. Although there is a great progress of therapeutic strategies targeting iron overload and ferroptosis in intestinal diseases, the mechanism of iron overload and ferroptosis in the pathogenesis of diseases has remained to be not fully clarified and designing effective drugs is a long-term project. Understanding the mechanisms will provide comprehensive strategies for the treatment and prevention of mucosal barrier dysfunction and intestinal diseases.

## Data Availability

Not applicable.
